# The Surgical Uses of Iodine

**Published:** 1911-02-25

**Authors:** 


### SURGERY.

### THE SURGICAL USES OF IODINE.

The value of iodine solution in disinfecting the skin before operation is now generally recognised and made use of by surgeons. Amongst the first to recommend it was Grossisch, and its introduction into this country was largely due to Mr. Thomas Bryant. Latterly Major F. J. W. Porter, who wrote a paper upon the subject in the first half of 1909, Mr. Waterhouse, and others have worked to reintroduce iodine to the notice of the profession, and the value of the method has been testified to by many surgeons since. Although it is most important that strict asepsis should be observed as stringently as with other methods, it has been discovered that by the use of iodine the preliminary washing, shaving, and preparatory dressing otherwise necessary before an operation can largely be done away with. The technique recommended is to paint the operation area with iodine solution: (1) one hour before the operation commences, (2) when the patient is on the table, and (3) when the operation is completed and the skin sutures are tied.

### Major Porter's Technique.

The painting of the skin area requires only a few seconds, and does away with a long and troublesome process, sometimes trying to the patient.

The preparation of iodine used is immaterial. Major Porter obtained good results with 10-per-cent. iodine in spirit. The majority of surgeons prefer a weaker solution to avoid setting up a dermatitis. Many solutions have been used—iodine 15 grains to chloroform 1 ounce; iodine 5 per cent., ether 5 per cent. dissolved in spirit. Wallace's alcohol and acetone iodine solution is not to be recommended. For ordinary surgical work a 2-per-cent. solution of iodine in spirit is usually satisfactory. Lieut. Mitchell's experience has been with the tincture of iodine, and he records his views in a recent paper in the *Journal* of the Royal Army Medical Corps. The skin area need not be shaved, and no matter how dirty the part is it should on no account be washed previous to the operation. The clefts between the cells of the skin contain fat, sweat, and bacteria; if the skin be washed with soap and water the cells swell up and prevent the iodine penetrating into these clefts. The advantages of adding a fat solvent, such as ether, to the iodine solution are evident. The iodine solution acts by fixing the bacteria, hardening the skin, and causing slight hyperæmia. (Continental observers, it may be added in parenthesis, do not lay stress on this point, which is probably not of real importance so far as technique is concerned.) In from seven to ten days there is a slight desquamation of the skin. The solution can be used on patients of all ages, even on the skin of infants and on that of the very aged.

### Advantages of the Method.

To sum up, the advantages of this method of skin disinfection are:—

(1) Efficiency, proved by the results published by Porter, 30 cases; Waterhouse, 150 cases; Fenwick, 110 cases; Stretton, 51 cases; König, 251 head injuries; Professor von Eiselsberg, 200 cases. Iodine sterilisation is now the routine method used in all German clinics.

Lieut. Mitchell, R.A.M.C., has used this method of skin disinfection in twenty-five cases, including operations for varicose veins, varicocele, tumour of female breast, removal of cysts, and severe wounds of the head, and has always obtained healing by first intention.

(2) Ease, quickness, and certainty of application; (3) the area of skin disinfected is mapped out; (4) there is no discomfort to the patient; (5) expense is slight; (6) the solution of iodine can be used in all emergency operations, and valuable time is not lost.

The operator's hands can be efficiently sterilised by washing them with ethereal or turpentine soap for five minutes, and then placing them for two minutes in a quart of water containing 1 drachm of tincture of iodine.

The vagina may be efficiently sterilised for gynæcological operations by plugging it with swabs soaked in iodine and spirit solution.

Iodine and spirit solution is a very useful dressing in septic wounds and discharging tubercular sinuses.

### Therapeutic Uses.

An important therapeutic though unknown use to which the tincture of iodine may be put is as an antidote to the caustic action of carbolic acid. Great relief is immediately obtained by soaking any external part injured by carbolic acid in a basin of water to which 1 drachm of tincture of iodine has been added. In the case of internal carbolic-acid poisoning, ½ to 1 drachm of tincture of iodine in half a pint of tepid water should be administered.

### Ligatures and Sutures.

The use of iodine in the sterilisation of catgut was first recommended by Claudius in 1902. Since that year the writings of Salkindsohn, Scott-Riddell, Stewart, Moschowitz, Dickie, and others have all shown iodised catgut to be the most efficient and cheapest catgut in use at the present time. To prepare the catgut all that is necessary is to place the commercial hanks in the following solution for eight days: Iodine 1 part, potassium iodine 1 part, water 100. The catgut, before use, is washed in a weak carbolic solution; the bottle in which it is stored should have a well-fitting lid, otherwise the iodine may evaporate. At the end of eight days the catgut is very dark in colour. Some of the hanks may become brittle and uneven, according to the quality of the catgut. Instead of using the watery solution, the catgut may be prepared by placing it in a solution of iodine 1 part, spirit 15 parts. When catgut is prepared by this method it may swell up and feel sodden when placed in a weak carbolic solution or normal saline. This defect can be overcome by storing the catgut in a 1-per-cent. solution of formalin after it has been prepared in iodine and spirit.

Catgut prepared in this way will last in the tissues for: No. 00, six days; No. 1, eight days; No. 2, ten days.

### Moschowitz's Method.

Moschowitz has gone further, and uses iodine-prepared catgut dry. His method is simple. After eight days in a watery solution of iodine he lifts the hanks with sterilised forceps, and places them in a sterilised glass dish fitted with a cover. In the bottom of this dish is a small beaker containing concentrated sulphuric acid. The sulphuric acid absorbs the moisture, and in a few days the catgut is dry. The advantages of handling dry catgut and the ease with which ligatures can be applied may be imagined, but cannot be fully appreciated until the dry catgut has been used.

After many trials Moschowitz has come to the following conclusions: (1) Dry iodine-prepared cat gut is absolutely sterile, and remains so for years; (2) it is impossible to infect it by ordinary means; (3) it does not act as an irritant in the tissues; (4) the tensile strength of dry iodised catgut is superior to wet; (5) it is very cheaply prepared; (6) it is absorbed when it has served its purpose. Silk, silkworm gut, and horsehair can all be similarly rendered sterile by means of iodine.

Lieut. Mitchell's experience of ligatures prepared by the iodine method extends over some years, and his results have always been satisfactory.

